# Treatment of unaccompanied minors in primary care clinics - caregivers' practice and knowledge

**DOI:** 10.1186/s13584-018-0217-0

**Published:** 2018-06-01

**Authors:** Maya Peled-Raz, Michal Perl, Manfred S. Green

**Affiliations:** 1 School of Public Health, The Center for Health, Law and Ethics, University of Haifa, Haifa, Israel; 2 Clalit Health Services, Haifa, Israel; 3 School of Public Health, University of Haifa, Haifa, Israel

**Keywords:** Consent to treatment, Children’s rights, Best interests of the minor, Parental notification

## Abstract

**Background:**

By law, the provision of medical treatment to minors in the State of Israel is conditional upon the consent of their parents. In 2004, the Head of the Medical Administration Unit in the Ministry of Health issued Circular No. 4/2004 regarding the treatment of un-accompanied minors in primary care clinics. This circular aims to expand on the law, and permits the treatment of certain minors without parental attendance or consent. The circular does indicate that parents should be notified of the treatment retroactively, and provides cases in which it is possible to avoid notification altogether.

The objectives of this study were: (a) to examine the scope of treatment of unaccompanied minors in primary care clinics; (b) to examine caregivers’ knowledge of the provisions of the law and of the circular; and (c) to examine the implementation of the law’s and the circular’s provisions relating to the treatment of unaccompanied minors in primary care clinics in the community.

**Methods:**

In a cross-sectional study, we surveyed 158 doctors and nurses from primary care clinics of the Haifa and Galilee districts of “Clalit Health Services”. Respondents were selected via a snowball method, with attention to ensuring a heterogeneous clientele and geographic dispersion.

**Results:**

Treatment seeking by unaccompanied minors is an existing and even widespread phenomenon. The vast majority of unaccompanied minors were in effect treated without parental consent. The main reason for minors’ solitary treatment seeking was parents being busy. In 40% of the cases, where minors were treated without the presence and consent of their parents – parents were not notified of the fact. None of the respondents correctly answered all questions regarding the relevant provisions of the law and circular, and only 10% answered all the questions regarding the circular’s parental notification requirements.

**Conclusions:**

The Israeli legal arrangement, pertaining to the provision of treatment to minors without the consent of their parents, is vague, unclear to medical and nursing practitioners and limited in terms of the needs of the minors themselves, as well as the needs of the medical system.

There is a need for methodical and coherent regulatory thinking on the subject, as well as more thorough education of both nurses and physicians, in order to ensure the rights and interests of minors as well as the rights of their parents.

**Electronic supplementary material:**

The online version of this article (10.1186/s13584-018-0217-0) contains supplementary material, which is available to authorized users.

## Background

The provision of medical treatment to minors (aged 0–18 year old) in the State of Israel is conditional upon the consent of their parents [1]. From this rule, the law precludes simple and ordinary treatment, which may be given where parents could not be located in a reasonable time frame [2], as well as urgent treatment, which is permissible (and even required) without the consent of the parent – both for the protection of the particular minor’s best interest and wellbeing [2]. In addition the Israeli law exempts two types of treatment from parental consent –minor’s pregnancy termination [3] and HIV testing [4] - mainly for the promotion of public health.

Extensive discussion has been conducted over the years – in the academic [5–7], professional [8, 9] and regulatory [10–12] spheres alike, about the limited scope of the exceptions to the parental consent requirements, and the need to expand them, in order to promote a range of goals, primarily the best interests of minors and the rights of minors seeking treatment. This discussion has succeeded in somewhat extending the legal recognition of the rights of minors to influence their treatment (for example in allowing a 16 year old minor to veto his genetic testing [13], as well as conditioning a 15+ year old patient’s psychiatric commitment on his additional assent [14] among others). However, the legal obligation to receive parental consent, for any treatment that does not fall into such exceptions, remained as comprehensive as ever.

In light of incomplete attempts to more comprehensibly regulate the treatment of minors and their consent to treatment, the Ministry of Health attempted to define rules of thumb that will help practitioners deal with the dissonance between regulation and the needs of their minor patients. Thus, in 2004, the Head of the Medical Administration Unit of the Ministry of Health issued Circular No. 4/2004 concerning “visits of minors to primary care clinic without the presence of their parents” (herein – the circular) [15]. The circular aims to expand on the law, and permit the treatment of certain minors without parental attendance or consent.

### The circular’s provisions

The purpose of the circular, as stated in its preamble, is to guide caregivers in primary care clinics, in the examination, delivery of a diagnosis, recommendations for further tests and treatment of minors in one of two conditions:

1. When a minor seeks treatment without an adult escort - alone or with another minor.
2. When the minor comes accompanied by an adult who is not his parent or guardian (grandfather, neighbor, older brother or other).

By doing so, so the circular’s preamble attests, it seeks first and foremost to protect the best interests of minors, by allowing the administration of care, when needed, in cases where the insistence on prior parental consent would serve as an obstacle to good healthcare delivery.

The guidelines in this circular do not prevent the caregiver from requesting parental consent for treatment in any case he deems appropriate. They also do not apply to circumstances where specific legal provisions relating to the treatment of minors exist. The guidelines apply only to caregivers (doctors, nurses and other caregivers) in primary care clinics in the community, and not in any other medical institution, and only to a minor and his/her family who are familiar to the medical staff in the clinic.

The Ministry of Health’s circular drafting committee found that minors aged 14 and over tend to turn to primary and routine medical treatment unaccompanied, and that they usually have the intellectual and mental ability to understand the information needed to make a decision and give informed consent to routine treatments.

Accordingly, the circular distinguishes between a minor who is over 14 years old and a minor who has not yet reached the age of 14 years. This distinction corresponds to, and relies on article 6 of the Legal Capacity and Guardianship Act, which allows a minor to preform legal actions, without the consent of his legal representative, “when these are actions that are commonly practices be minors of his age”.

Nevertheless, the circular states that when the caregiver feels that the minor is not emotionally and mentally mature as is expected of his age, he will act as if the minor was not yet 14 years old.

The circular attempts to minimize infringing on parental guardianship, and states that “the permission to obtain informed consent from a minor over the age of 14 does not intend to lessen the authority of the parents, who have full responsibility and authority over the minor until the age of 18”. To that aim, the circular demands that a summary of the minor’s medical examination and treatment be at least given to the minor in writing for delivery to his parents (and in some cases a more prompt phone call to the parents would be in order). However, the circular allows the caregiver to act without the knowledge of the parents in cases where he/she assesses that parental involvement may harm a minor or when the minor strongly opposes his parents’ involvement in treatment. In such cases, the caregiver must involve a Welfare Officer. For minors who are under the age of 14, according to the circular, the consent of a parent, verbally or in writing, is required.

As the application of the circular and caregivers familiarity with it have hardly been researched, we designed a survey with three objectives in mind: (a) to empirically examine the characteristics of the unaccompanied minors seeking treatment (here after unaccompanied minors or UAMs) phenomena in primary care clinics; (b) to examine the acquaintance of relevant caregivers with the provisions of the law and of the circular; and (c) to examine the implementation of the law’s and circular’s provisions as they relate to the treatment of minors in primary care clinics in the community.

## Methods

A total of 158 Israeli physicians and nurses, from primary care clinics of the Haifa and Galilee districts of “Clalit Health Services” HMO, were anonymously surveyed using snow-ball method – some by a collected hard-copy survey and some by Google Docs platform. This group was chosen to be surveyed for its heterogeneous cliental and vast geographical dispersion.

The survey included questions regarding respondents demographics, specialty, primary cliental (rural/urban, Jewish/Arab/mixed, socio-economic status) their actual experience with UAMs, their knowledge of the provisions of the Israeli law and the Ministry of Health’s circular and on whether they had gone through any relevant training. Participants’ acquaintance with the Law’s and circular’s provisions where evaluated using short hypothetical scenarios. For 10 scenarios, respondents were asked to indicate whether the treatment of the described UAM was allowed or prohibited, sans parental consent. For 6 more scenarios, respondents were asked to indicate whether it was allowed not to (at least retroactively) notify the parents about the minor’s condition and treatment. The correct answers to each scenario were determined by the expert opinion of 2 medico-legal experts. The questions and scenarios are listed in Additional file 1: Appendix.

Statistical analysis was done by a professional statistician using SPSS Statistics software, Version 22.

## Results

One hundred fifty eight questionnaires were analyzed. 65% of respondents were nurses. 35% were physicians, of them 24 were pediatricians (15.2%), 13 family physicians (8.2%), 10 general practitioners (6.3%) and 5 pediatric or family-medicine residents (3.2%). 80.4% (n 127) of respondents were female; their average age was 47y (±9.09); 67.7% (n 107) were born in Israel and 15.8% (n 25) in the former USSR and Eastern Europe; 80.4% (n 127) completed their professional studies in Israel.

Most respondents work in an urban set clinic (77.8%, n 123), serving mostly a Jewish population (66.5% n 105) or a mixed Arab and Jewish populations (27.2% n 43). 46.8% (n 74) of respondents assessed their patients’ socio-economic status as medium-low, while 31% described their patients as from a medium-high socio-economic background, 15.8 (n 25) as from a low socio-economic background and 5.1% (n 8) testified that they were mostly serving a high socio-economic population.

The response rate veered around 32% (out of 500 questionnaires distributed by hard copy – which served as the central surveying method), and a relative small number of respondents came from rural, predominantly Arab community serving clinics (n.3) – a fact that limits the relevant statistical analysis in our research as well as its generalizability.

### Encounters with minors not accompanied by their parents

As described in Tables 1, 74.1% of respondents testified that they were asked to treat UAMs in the past year. When asked to estimate the occurrence of UAM in their clinic, most (58.2%, n92) estimated UAMs comprise less than 10% of treated minors, while 16.5% estimated them to be between 10 and 25% of cases, 4.4% believed they comprised 25–50% of the cases and 5 of the respondents (3.2%) testified to over 50% of their minor patients coming in unaccompanied. Surprisingly, no significant differences were found between rural and urban clinics in minors’ tendency to seek treatment un-accompanied (93.1% v. 84.3% respectively encountered the phenomenon in the last year, *p* = 0.225); nor between clinics that serve mostly Arab, Jewish or mixed populations (all between 82%–87.5%). Socio-economic status was also not found as a significant factor in parental attendance. This lack of significance should be at least partly attributed to the small sample of rural and exclusively Arab-population serving clinics.

**Table 1:** Occurrence of Un-Accompanied Minors (UAM) seeking treatment

		%	N (/out of reference group)	
Encountered UAM in the last year	total	74.1	117/158	
UAM out of all minor patients	Less than 10%	58.2	92	
	10–25%	16.5	26	
	25–50%	4.4	7	
	More than 50%	3.2	5	
	DNA	17.7	28	
Encounter with UAM by socio-economic background	low	95	19/20	Chi-Square 4.422, *P* = 0.219
	Low-medium	80.6	50/62	
	Medium high	90.9	40/44	
	High	75	6/8	
Encounter with UAM by clinic’s cliental	Arab	87.5	7/8	
	Jewish	87.4	76/87	
	Mixed	82	32/39	
Encounter UAM by clinics location	Rural	93.1	27/29	*P* = 0.227
	Urban	84.3	86/102	

The vast majority of UMAs were in-effect treated without parental consent (67% if respondent testified that they refused treatment of UMA in less than 10% of such cases).

It’s important to note, that most UAMs where in fact accompanied by someone – most commonly by grandparents (over 60% of respondents indicated that that was the most common scenario). Only 24.2% (n31) thought that minors usually came in alone, when not accompanied by their parents.

When asked as to the most common reasons for the lack of parental presence by the minor’s side, respondents pointed to the parents being busy as the prominent reason (37% testified to it as being common or very common). About 35% pointed to a long-standing acquaintance between the minor, his family and the care-taker as a recurring reason and 25% identified the parents’ perception of the minor as mature enough as a central factor (common or most common). Lack of parental knowledge and the minor’s preference not to be accompanied were perceived as un-common or rare motivations (83% and 91.5% respectively viewed it as such).

Lack of parental knowledge seems to affect Arab-centered clinics more than others (33.4% of participants from such clinics testified that it is a common or very common reason, as compared to 12% in the predominantly Jewish-population serving clinics); yet no significance can be attributed to that effect. Also, interestingly, parents being busy was perceived as a rarer motivator (for lack of parental presence) in urban clinics, though the small sample of rural clinics (n29/152) did not allow for significance to be measured.

### Application of documentation and notification requirements

Sixty nine percent (n 109) of respondents testified that they document the lack of parental presence and consent either always or in most cases, and 51.2% testified to also documenting the identity of whoever was present in parents’ stead. 15.8% never or usually don’t document any of it, and a staggering 15% (n 24) chose not to answer the question at all. More importantly, although the circular requires notification of parents in cases where a minor is examined and treated unaccompanied, only about 60% of respondents declared that they notify parents retroactively – always or most of the time – about their child’s condition and treatment (usually by a phone call). 40% of respondents treat minors without making sure their parents are aware of their medical needs and treatment.

### Acquaintance with the law’s and circular’s provisions

Question 14 of the questioner included 10 scenarios – listed in Additional file 1: Appendix. Participants were asked to indicate in which of them it was legally allowed to treat a minor without the presence and/or consent of his or her parents – whether based on the law’s provisions or on the circular’s.

None of the participants responded correctly to all the scenarios, and the average number of correct answers stood on 6 out of 10 (with number of correct answers ranging between 2 and 9).

Physicians were found to be more knowledgeable in issues relating to pregnancy termination (Mann-Whitney U = 1953, *p* = 0.019). while nurses showed more command of the questions relating to prescription of birth control pills (Mann-Whitney U = 1872, *p* = 0.020) and to giving a 17 year old authorization to exercise at the gym (Mann-Whitney U = 2033.5, *p* = 0.018). In all other scenarios - both sectors showed equal partial knowledge.

Question 15 of the questioner included 6 scenarios – also listed in Additional file 1: Appendix, aimed at assessing respondents commend of when it was permitted to not notify parents of their child’s treatment seeking.

Only 3.8% of physicians (2/52) and 13.6% of nurses (14/103) – merging into 10% of total respondents – correctly reacted to all six scenarios, in most cases with no significant difference between the two sectors. The average number of correct answers stood on 4 out of 6 (with number of correct answers ranging between 1 and 6).

It has been shown in the study that there is a direct correlation between receiving training on the subject and the level of knowledge regarding treatment of minors, yet regrettably, only 41.1% of the respondents claimed that they were in effect trained on this important subject.

## Discussion

Our Study points to a substantial incidence rate of UAMs. It is important to note that the incidence rate is based of respondents’ estimation, and not on medical files review. Another limitation to our study, which may only be remedied by a systematic medical files review, is the fact that we did not attempt to collected data regarding the type of medical problems UAMs presented with (both in-of-themselves and as compared to accompanied minors).

The most significant finding of the study, in our opinion, relates to caretakers’ lack of familiarity with the current legal situation in Israel. Also, surprisingly, we saw that in many cases, more nurses correctly responded to scenarios, in which treatment is the sole authority of physicians – and vice versa.

A clear and potent example of both these observations may be found in the scenario describing a minor seeking referral to an HIV antibodies test. The Israeli law has authorized the referral of minors (aged 14 and up) to a blood test for HIV antibodies without the knowledge or consent of their parents back in 1996 [4]. In 2016, some twenty years after the enactment of said authorizing regulation, only 52% of respondents were aware of this. That is, almost half of them incorrectly answered the question and do not know what the position of the law is in this case. Furthermore, 94% of nurses answered this question correctly, even though they are not the ones who give referral for testing.

Also, the Israeli Penal Law of 1977 has since its enactment permitted pregnancy termination at any age, without the knowledge of the girl’s parents – if she so chooses. In this case (scenario 14.7), too, 47% of respondents wrongly appraised the legal stance and about 10% claimed that they did not know the answer.

Respondents showed greater knowledge of the Israeli law’s emergency exemption to parental consent – as 89.2% of respondents correctly answered scenario 14.5 – describing a 7 year old UAM, seeking treatment after a fall trauma, while accompanied by his school teacher.

Our findings correspond with the findings of a study conducted in primary care clinics in the Southern District of “Clalit Health Services” in 2008 – The only other study that had ever been conducted on this issue in Israel. In that study approximately 50% of respondents – all physicians, incorrectly answered the questions relating to the understanding and implementation of the arrangement regarding the treatment of UAMs [16].

While that early study – conducted only 4 years after the circular’s issue, could be viewed as attesting to a slow implementation process, our study’s findings can no longer be attributed to that.

The data collected in this study reinforces the feeling that the issue of treatment of minors involves considerable complexity and suffers from caretakers’ systematic lack of familiarity with the legal arrangements that regulate it.

We believe that our study’s results should be generally attributed to the sporadic and non-consistent nature of the Israeli regulation of the treatment of UAMs, and minors in general, as well as specifically ascribed to the complex wording of the 2004 circular [17].

As described before, due to the Israeli parliament’s lack of success in coherently regulating the statues of mature minors in treatment settings, it retorted to anecdotal solutions, accompanied by somewhat ambiguous and limited Ministry of Health’s professional guidelines. These solutions further complicate the legal state of affairs, confuse the treating staff, and in many cases place caregivers in absurd situations.

For example, as described above, there is no age restriction and it is legally permitted in Israel to terminate the pregnancy of an assenting minor without informing her parents. However, it is generally forbidden to subscribe the use of contraceptives to a UAM, without her parents’ permission (unless the minor is well known to the physician, and the prescription is accompanied by retrospective parental notification; or according to a new Ministry of Health’s circular – if the minor has already undergone an abortion in the past [18]). It is likewise officially forbidden to treat other serious sexually transmitted diseases, such as herpes or syphilis, which cause severe pain and mental distress. Ironically, 16-year-olds may serve as volunteer paramedics, as part of their school assignments, making medical decisions and caring for others, while in many situations they cannot make medical decisions regarding their own selves.

Such complex, vague and paradoxical legal conditions may lead to mistaken judgment, impede caregivers, increase legal litigation, and most importantly - may prevent adolescents from seeking and receiving well needed treatment.

The value of involving children and adolescents in their own medical decision-making is increasingly recognized around the world [19, 20], and minors have been shown both in Israel and abroad to seek health care unaccompanied in non-negligible numbers. [21] Yet, Contrary to the legal trend in other western countries,^1^ Israeli law has not yet managed to properly accommodate these times and needs – at all, and as our research shows – at least not in an applicable way.

The goal to strive for is to allow for a coherent yet age-flexible legislation, adapt to changing times, while maintaining parental authority.

Inspiration for such a coherent regulatory scheme may be found in Canada, in which (with the exception of the province of Québec) the determining factor in a child’s ability to provide or refuse consent is whether the young person’s physical, mental, and emotional development allows for a full appreciation of the nature and consequences of the proposed treatment or lack of treatment — and not whether or not the person has attained the age of majority. [22] in some of the Canadian provinces a default has been set, indicating ability to consent over a certain age, yet a younger person may still have the legal authority to consent, with no need for parental approval, if in the opinion of a legally qualified medical practitioner, he or she is capable of understanding the nature and consequences of the treatment and the treatment is in his or her best interests [23].

As long as such coherent legislation cannot be achieved, we recommend that the Ministry of Health’s circular be Simplified. Also, in light of the findings, which showed that trained teams where more knowledgeable of the legal requirements – it is important to train the relevant teams on the treatment of UAMs and provide them with tools to help them in future dilemmas.

## Conclusions

The Israeli legal arrangement, pertaining to the provision of treatment to minors without the consent of their parents, is vague, unclear to medical and nursing practitioners and limited in terms of the needs of the minors themselves, as well as the needs of the medical system.

In order to properly serve the health needs of minor patients, there is a need for a thorough rethinking and rewriting of the present legal stance on the delivery of minor’s medical care in Israel. Till such coherent changes are made, the MoH and its partners (mainly Israeli HMO’s) should invest in the thorough and in-depth training of health care practitioners and assist them in relevant decision-making processes.

## Additional file

Additional file 1: Question 14: According to the existing law in Israel today, in which of the following scenarios is it permitted to examine and treat a minor without the prior consent of a parent?. Question 15: In which of the following circumstances is it legally permitted not to inform the parents of the fact that the minor has been examined and/or treated without their presence?. (DOCX 16 kb)

## Abbreviations

HMO: Health Maintenance Organization (aka Kupat Holim)
MoH: Ministry of Health
UAM: Unaccompanied minors

## Footnotes

1 For example, In the UK, Minors older than 16 years old may consent to their own medical care. Also, according to common law, there are circumstances in which minors under 16 years of age could consent to their own medical treatment. In order to do so, the child or young person must have a ‘sufficient understanding and intelligence to enable him or her to fully understand what is proposed’. The level of maturity required to provide consent will vary with the nature and complexity of the medical treatment. (Gillick v West Norfolk and Wisbech Health Authority [1986] 1 AC 112.). These principles, as established in Gillick, were also endorsed as part of Australian common law (Secretary, Department of Health and Community Services v JWB and SMB (1992) 175 CLR 218). See also Bird S., Consent to medical treatment: the mature minor, Australian Family Physician 2011; 40(3): 159.
