# Supplementary material for: Treatment of unaccompanied minors in primary care clinics - caregivers' practice and knowledge
Source: Isr J Health Policy Res. 2018 Jun 1;7:29. doi: 10.1186/s13584-018-0217-0 (PMC5984380; doi:10.1186/s13584-018-0217-0)
Supplement: Supplementary file 1 — Question 14: According to the existing law in Israel today, in which of the following scenarios is it permitted to examine and treat a minor without the prior consent of a parent?. Question 15: In which of the following circumstances is it legally permitted not to inform the parents of the fact that the minor has been examined and/or treated without their presence?. (DOCX 16 kb) [file 13584_2018_217_MOESM1_ESM.docx]

**Appendix 1**

**Question 14:** According to the existing law in Israel today, in which of the following scenarios is it permitted to examine and treat a minor without the prior consent of a parent? *

|  |  | Yes | No | I do not know |
| --- | --- | --- | --- | --- |
| 14.1 | A 16-year-old girl who wishes to undergo a pregnancy test. | X |  |  |
| 14.2 | A 12.5-year-old boy who comes with his 17-year-old brother to the clinic for blood tests. | X |  |  |
| 14.3 | A 13-year-old girl, known to the family doctor, who comes with sore throat without fever. |  | X |  |
| 14.4 | A 15-year-old girl who is not familiar to the doctor, who comes in requesting a prescription for oral contraceptives. |  | X |  |
| 14.5 | A 7-year-old boy who was brought by an accompanying teacher after he fell and was injured in the head during the break at school. | X |  |  |
| 14.6 | A 16-year-old boy requesting a referral for a blood test to identify HIV antibodies. | X |  |  |
| 14.7 | A 13-year-old girl who wants to have an abortion. | X |  |  |
| 14.8 | A 17-year-old girl who asks the family doctor for permission to practice in a gym. | X |  |  |
| 14.9 | A 13-year-old boy who arrives at the clinic to have his dressing changed following an injury he suffered a week ago. |  | X |  |
| 14.10 | A 10-year-old boy who comes to the doctor with his 15-year-old brother due to ear-aches, with a note from the parents, allowing the brother to make decisions about treatment for his younger brother. | X |  |  |

**Question 15:** In which of the following circumstances is it legally permitted not to inform the parents of the fact that the minor has been examined and/or treated without their presence?*

|  |  | Yes | No | I do not know |
| --- | --- | --- | --- | --- |
| 15.1 | The minor refuses to inform his parents, and a Welfare Officer has been informed | X |  |  |
| 15.2 | The health care personal believes the minor's parents endanger the minor, and a Welfare Officer has been informed | X |  |  |
| 15.3 | The minor is mature enough and does not need accompaniment. |  | X |  |
| 15.4 | The minor came in accompanied by a responsible adult, such as a grandfather / grandmother. |  | X |  |
| 15.5 | Locating the parents to inform them requires an unreasonable investment of time |  | X |  |
| 15.6 | Treatment given is simple and trivial |  | X |  |

* *The correct answer is tagged with an X, for the convenience of the reader.*
